# Technique for continuous bedside monitoring of the global cerebral energy state

**DOI:** 10.1186/cc14532

**Published:** 2015-03-16

**Authors:** R Jakobsen, A Granfeldt, TH Nielsen, P Toft, CH Nordström

**Affiliations:** 1 Odense University Hospital, Odense, Denmark; 2 Regional Hospital of Randers, Denmark

## Introduction

In the present experimental study we explore whether cerebral venous lactate/pyruvate ratio (LP ratio) measured by intravascular microdialysis during induced hemorrhagic shock may be used as a surrogate marker for compromised cerebral oxidative metabolism.

## Methods

Six female pigs were anesthetized and vital parameters was recorded. Microdialysis catheters were placed in cerebral hemisphere parenchyma, the superior sagittal sinus and femoral artery. Brain tissue oxygenation (PbtO_2_) and intracranial pressure (ICP) was recorded. Hemorrhagic shock was achieved by bleeding the animals to a mean arterial pressure (MAP) of approximately 35 mmHg. Animals were kept at a MAP of about 30 to 40 mmHg for 90 minutes. The animals were resuscitated with reinfusion of shed blood followed by 3 hours of observation.

## Results

In the cerebral hemisphere, hemorrhagic shock caused a marked increase in the LP ratio, while a significant but minor increase was observed in the sagittal sinus. The LP ratio increased and continued doing so to a very high level. In the femoral artery, the shock period was associated with a slight increase of the LP ratio. The increase in the LP ratio in the sagittal sinus was markedly and significantly higher than in the arterial blood. Further, the dynamic changes in the LP ratio in the sagittal sinus followed that of the parenchyma, not the arterial blood. After infusion of blood ICP increased, cerebral perfusion pressure and PbtO_2_ decreased and the microdialysis showed continuous signs of ischemia and cellular degradation.

## Conclusion

This experimental study documents that during protracted pronounced hemorrhagic shock, cerebral energy metabolism was severely compromised and exhibited a biochemical pattern typical of ischemia and cellular degradation. After retransfusion this pattern continued. From intravenous microdialysis in the sagittal sinus, it is possible to achieve semiquantitative information of the intracerebral redox state. Accordingly, it might be possible to monitor the global cerebral energy state continuously with a strictly extracerebral technique. This technique might be valuable in various severe conditions during critical care when cerebral energy metabolism may be compromised; for example, resuscitation after cardiac standstill, open heart surgery, multitrauma and so forth. Interestingly the study also showed that after reinfusion of blood other parts of the body recovered, evaluated by microdialysis, but the brain showed signs of damage, making the brain the limiting organ in hemorrhagic shock.

